# Ocular emergencies in the South Asia region

**Published:** 2019-02-10

**Authors:** Tarjani Makwana, Noopur Gupta, Praveen Vashist

**Affiliations:** 1 Senior Resident: Dr Rajendra Prasad Centre for Ophthalmic Sciences, All India Institute of Medical Sciences, New Delhi; 2 Assistant Professor: Dr Rajendra Prasad Centre for Ophthalmic Sciences, All India Institute of Medical Sciences, New Delhi; 3 Professor of Community Ophthalmology: Dr Rajendra Prasad Centre for Ophthalmic Sciences, All India Institute of Medical Sciences, New Delhi

**Eye care providers at different levels in South Asia must be able to diagnose, manage, initiate first-aid and refer during an ocular emergency.**

Ocular emergencies are an important cause of morbidity in South Asia and studying their spectrum and presentation is vital for developing local preventive and therapeutic programmes. Primary care providers must be able to diagnose, manage, initiate first-aid, or refer, as any delay in treatment during an ocular emergency can result in permanent loss of vision.^1^

## Ocular trauma

**Figure 1:**
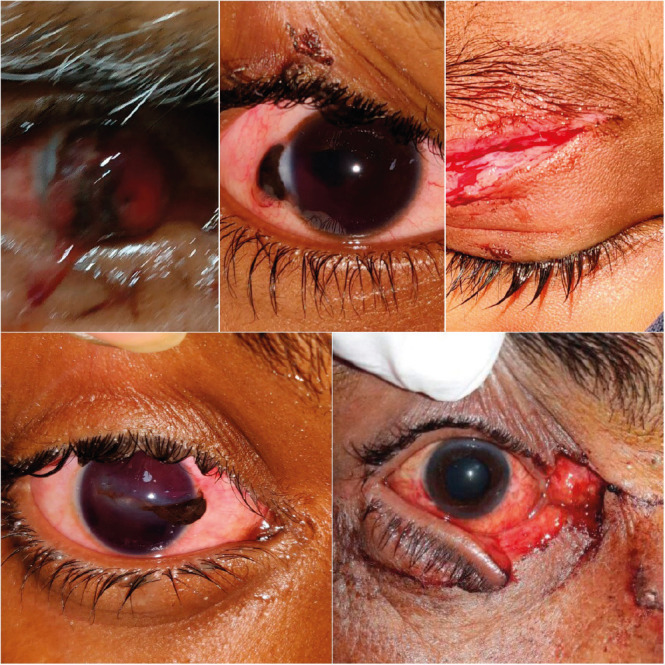
Mechanical injuries

Any form of trauma is an emergency and prompt treatment can arrest complications and long-term morbidity (Figure 1). The prognosis of any injury is commonly made worse by delayed presentation and use of inappropriate, untested products and traditional medicines.^2^ Health promotion interventions in injury prevention include raising awareness and actively involving the community. Workplace trauma can be prevented through occupational health laws which educate workers and promote the use of protective eyewear. Children are often victims of ocular trauma, so health education in schools is very important.

Ocular trauma can be classified into

- Penetrating injuries
- Blunt injuries
- Chemical injuries
- Ocular burns

### Penetrating injuries

Open globe injuries are caused by sharp objects in which there is full thickness wound in the eyewall.

The patient may present with a sudden loss of vision, pain, watering and an inability to open the eye. Visual acuity should be measured for each patient. Surgical closure is necessary in case of open globe injuries in order to minimise the risk of further infection. Intra-ocular foreign bodies, if present, should be removed; this requires specialist facilities and surgery.

### Blunt injuries

Closed-globe injuries are caused by blunt objects, where there is no full thickness wound of the eyewall comprising sclera and cornea.

The patient may present with loss of vision, pain and inability to open the eye. Visual acuity, pupillary reactions and the posterior segment should be evaluated in all cases. The management will depend on the severity of the injury. With conservative treatment, a simple hyphema will usually reabsorb after a few days.

### Chemical injuries

**Figure 2:**
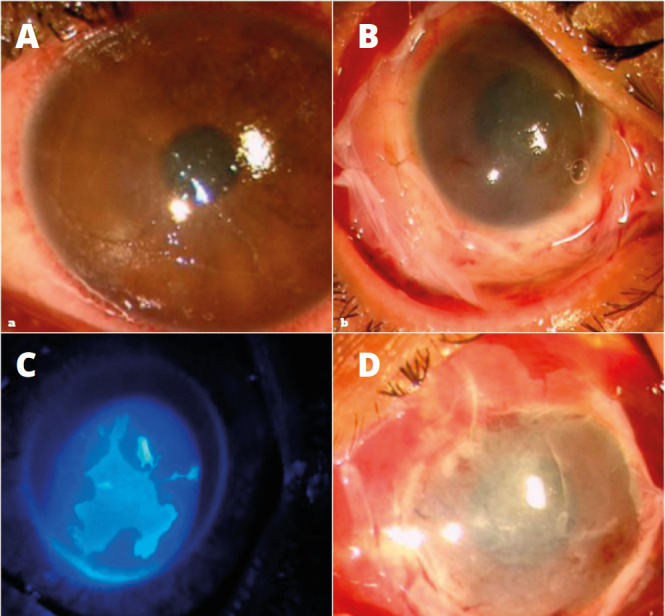
Chemical injuries of the ocular surface. **A.** Epithelial defect following acute chemical injury. **B.** Amniotic membrane transplantation **C.** Epithelial defect as seen on slit lamp under cobalt filter after fluorescein staining **D.** Amniotic membrane transplantation.

Chemical injuries may present in different ways, depending on the nature of the chemical agent, its concentration and volume, and the duration of exposure.^3^ Both acids and alkalis can cause eye injuries. Many occur in men who are at risk of exposure to chemicals such as lime (calcium hydroxide), ammonia, sodium or magnesium hydroxide in the workplace (Figure 2).

The first step in the management of chemical injuries is immediate and meticulous irrigation of the eye. This is done by everting the eyelids and flushing with ringer lactate or normal saline until the pH of the ocular surface is neutralised. Timely treatment that includes topical antibiotics, cycloplegics, topical steroids, topical sodium ascorbate & citrate 10%, oral doxycycline, oral ascorbate and tear substitutes must be instituted.

### Ocular burns

Ocular damage from thermal burns can result from contact with boiling liquid, molten metal, flames, gasoline explosions, steam or hot tar. Firecrackers can cause combined chemical and thermal burns on the ocular surface.

The management of ocular burns depends on the type of injury. However immediate cleaning and irrigation with normal saline or clean water is an important first aid measure.

### Corneal Ulcer

Corneal ulcers are common in the South Asian region, especially in countries with rural and developing economies.^4^ A corneal ulcer is defined as a corneal epithelial defect with infiltration of the deeper stroma, most commonly caused by infection. Viral ulcers arise spontaneously on a previously intact epithelium, while bacterial and fungal ulcers occur after a traumatic break in the corneal epithelium. Fungal ulcers typically start after an injury with organic matter.

Patients with a corneal ulcer present with pain in the eyes, foreign body sensation, photophobia, discharge, watering and blurred vision. It is important to elicit a proper history and sequence of events. Patients should be asked about ocular medications, especially the use of corticosteroids, previous eye surgery, ocular disease and systemic illness.

**Figure 3:**
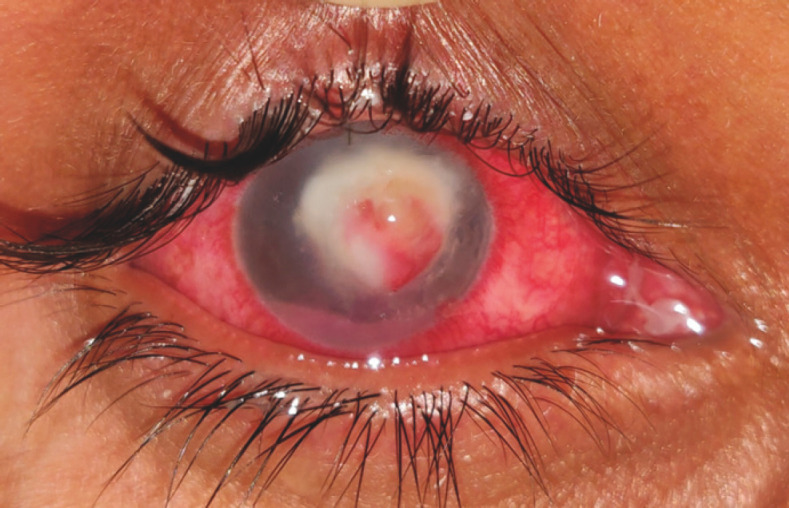
Fungal corneal ulcer

On examination, the eye will typically look congested with a white corneal lesion indicating stromal infiltration (Figure 3). A corneal scraping can be taken and sent for Gram and KOH staining along with bacterial and fungal culture and sensitivities, since determining the infectious aetiology is important to guide future treatment.

**Figure 4:**
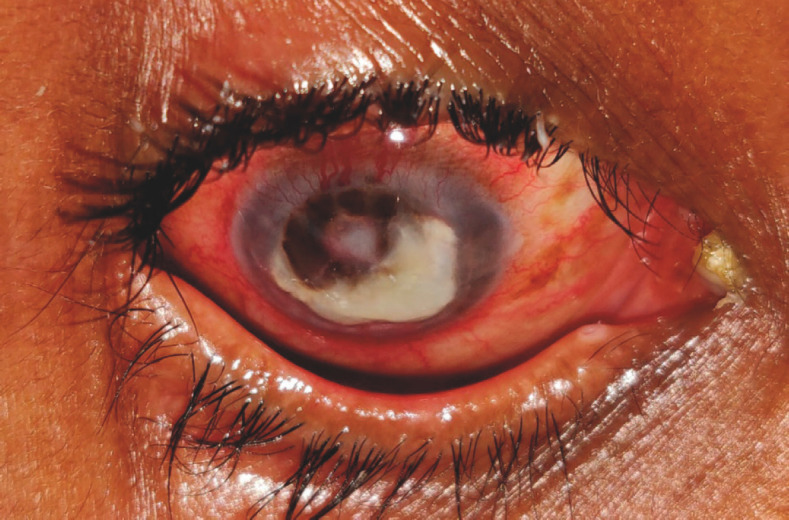
A penetrating corneal ulcer with sloughing of the cornea and uveal tissue show requiring urgent keratoplasty

**Figure 5:**
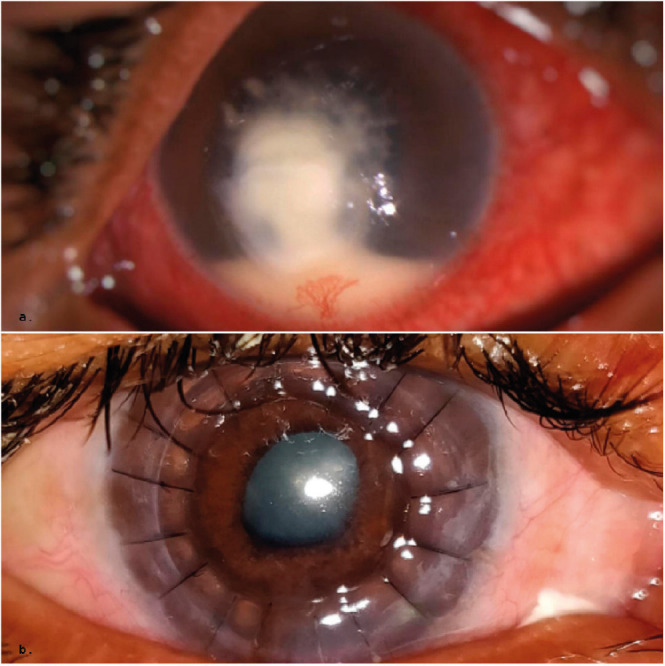
**A.** Fungal corneal ulcer with hypopyon **B.** Following therapeutic keratoplasty for the ulcer

Immediate initiation of a topical antibiotic followed by prompt referral to a higher centre is necessary. Fortified antibiotics such as tobramycin and a cephalosporin or vancomycin are appropriate for severe, deep, or central corneal ulcers. Fungal ulcers are treated with topical natamycin 5% or topical voriconazole 1% eyedrops. Supportive treatment like cycloplegics, oral analgesics and antiglaucoma agents maybe required. Close follow-up is essential for all corneal ulcers as non-resolving ulcers or penetrating ulcers (Figure 4) may require an urgent therapeutic keratoplasty to debulk the cornea of infectious tissue and/or restore the integrity of the eye (Figure 5).

### Prevention

Agricultural workers can use protective goggles that can aid in prevention of corneal ulcers. Community awareness of risk factors and effects of using traditional medicine can help in minimising severe consequences. Early recognition of symptoms, institution of appropriate treatment by the community health workers or ophthalmologists and prompt referral where necessary are critical in prevention of corneal ulcers.

### Acute glaucoma

Acute angle-closure glaucoma is caused by the sudden closure of the anterior chamber angle. This leads to inadequate drainage of the aqueous humour and a subsequent elevation in intraocular pressure (IOP) which can lead to optic nerve damage. It is more common in the South East Asia region and if not recognised and treated on time can cause blindness within hours.

Patients present with severe ocular pain, decreased vision, nausea and vomiting, intermittent blurring of vision with halos, and headache. Ocular examination shows conjunctival infection, corneal oedema, a mid-dilated pupil that does not react well to light, shallow anterior chamber and decreased vision. IOP usually ranges from 40 to 90 mm Hg.

Once acute angle closure is suspected, IOP is lowered with oral acetazolamide and topical timolol, pilocarpine, and apraclonidine, while monitoring changes to the angle and optic nerve head. Hyperosmotic agents such as oral glycerol or intravenous mannitol are effective in lowering IOP during an emergency. Once IOP is controlled laser iridotomy is performed in both the affected eye and the fellow eye as well to prevent acute attacks. Prompt, appropriate diagnosis, aggressive treatment and management are necessary to prevent, or minimise, significant ocular morbidity in patients with angle closure glaucoma.

## Acute loss of vision

Acute loss of vision in a white eye can occur due to central retinal artery occlusion (CRAO), retinal detachment, optic neuritis (Table 1). Immediate evaluation and referral to a tertiary care centre is important. Risk factors for CRAO include old age, being male, smoking, hypertension, diabetes, cardiovascular diseases and coagulopathies. Control of modifiable risk factors via health education and health promotion is the primary prevention of CRAO.

**Table 1:** Eye emergencies and care at different levels

Eye emergency	Primary level	Secondary Level	Tertiary level
**Penetrating injury**	History and examination Injection tetanus toxoid Start oral antibiotics Shield/protect the eye Refer to higher centre	History and examination Admission and urgent primary surgical repair under general anaesthesia Refer to higher centre if facility is not available	Surgical repair Intraocular foreign body removal by specialist Post-operative rehabilitation
**Chemical injury**	History Irrigation of the eye ++++ Urgent referral to higher centre	History + examination Irrigation of the eye ++++ Remove any particulate matter Start topical antibiotics, cycloplegics, and oral ascorbate Urgent referral to higher centre if indicated	Management depends on the severity and type of chemical injury. Severe alkaline burns may require long-term medical and surgical treatment
**Corneal ulcer**	History and examination Topical antibiotics e.g. eye ointment chloramphenicol 1% **NEVER START STEROIDS** **Stay away from traditional eye medicines** **Refer** to a higher centre	History + examination Confirm diagnosis of corneal ulcer Take corneal scraping for KOH/ Gram smear to identify organism Admit the patient if facility is available, if there is a threat to vision and to ensure treatment compliance and follow-up **Refer to a tertiary ophthalmic centre if:** the patient is a child there is impending or actual penetration it is in the only functional eye no facility for corneal scraping **TREATMENT:** *NO FUNGAL HYPHAE*- Start topical cefazolin 5% and tobramycin 1.3% hourly If no improvement after three days **REFER** *FUNGAL HYPHAE seen* – Start topical natamycin 5% If no improvement after three days **REFER**	History + examination Confirm diagnosis and classification Take corneal scraping for smear and culture for antibiotic and anti-fungal sensitivity Admit if indicated Initiate empirical treatment and then targeted treatment based on microbiology workup Systemic antifungals are recommended in fungal ulcers, which are: large and deep, associated hypopyon, perforating, or have scleral involvement Systemic antibiotics are recommended in bacterial ulcers if there is scleral involvement, associated endophthalmitis or perforation If responding to treatment, taper frequency of drops and follow up If no response to treatment or perforation, consider: **SURGICAL OPTIONS** Surgical debridement Tarsorrhaphy Patch grafts Conjunctival flaps Penetrating/lamellar keratoplasty
**Endophthalmitis** History of intraocular surgery or trauma Redness, pain, watering, lid edema Decreased visual acuity Hypopyon may be present in most cases.	Urgent referral to tertiary centre	Urgent referral to tertiary centre Start topical and oral antibiotics (fluoroquinolones)	Vitreous tap Systemic antibiotics Intravitreal antibiotics *Vancomycin 1.0 mg/0.1 mL* *Ceftazidime 2.25 mg/0.1 mL* *Amphotericin B 5-10 ug/0.1 mL* (if fungal suspected) Corticosteroids (to modulate the ongoing host inflammatory response), avoid when fungal is suspected Pars plana vitrectomy if no response to treatment
**Orbital cellulitis** Fever Lid oedema Proptosis Painful ocular movements Decreased visual acuity	Urgent referral to tertiary centre Start oral antibiotics	Urgent referral to tertiary centre Start IV / IM or oral antibiotics	Admission Blood culture and routine blood tests Orbital imaging will reveal pus pockets in the orbit / infection in the paranasal sinuses Intravenous broad-spectrum antibiotics for initial seven days followed by shifting to oral antibiotics for seven-14 days. I.V. Vancomycin 40 mg/kg/day in 2-3 divided doses per day I.V. Ceftriaxone 100 mg/kg/day in 2 divided doses per day I.V. metronidazole 30 mg/kg/day in 3 divided doses per day (in case of suspected anaerobic organism) Occasionally surgical drainage of orbital abscess by ophthalmologist and surgical drainage of sinuses by ENT specialist
**Acute glaucoma** Sudden onset, unilateral ocular pain Headache Coloured halos Decreased visual acuity Nausea and vomiting	Immediate recognition Check intraocular pressure digitally (eye will be stony hard) or with *TONOPEN* Give oral acetazolamide 500 mg stat Urgent referral to higher centre	Check intraocular pressure Oral acetazolamide 500 mg Topical beta blocker (timolol) Admission if facility available Hyperosmotic agents (intravenous mannitol or oral glycerine) Laser PI if available	Check intraocular pressure Oral acetazolamide 500 mg Topical beta blocker (timolol) and / or 2% piolocarpine Hyperosmotic agents (intravenous mannitol or oral glycerine) Gonioscopy to check angle Laser PI Consider glaucoma filtering surgery once acute stage is managed
**Optic neuritis** Unilateral visual loss Pain with ocular movements Afferent pupillary defect Colour vision deficiency Disc may be normal (retrobulbar neuritis) or swollen (papillitis) Visual field loss	Urgent referral to tertiary centre	Urgent referral to tertiary centre	Confirm diagnosis Complete blood investigations. Start ***intravenous high-dose methylprednisolone for three days followed by 11 days of oral 1 mg/kg/day prednisone*** after tuberculosis and malignancy is ruled out. Neurology referral with MRI brain
**Retinal detachment** Sudden onset, painless loss of vision Flash of light, floaters and curtain falling in front of eye	Urgent referral to tertiary centre	Perform dilated fundus examination/fundus camera Urgent referral to tertiary centre	Immediate surgical management by a vitreo-retina specialist Screening of the other eye for any predisposing lesions and prompt laser.
